# Incidence and characteristics of aspiration pneumonia in the Nagasaki Prefecture from 2005 to 2019

**DOI:** 10.1186/s12890-024-03015-8

**Published:** 2024-04-20

**Authors:** Iku Tomonaga, Hironobu Koseki, Chieko Imai, Takayuki Shida, Yuta Nishiyama, Daisuke Yoshida, Seiichi Yokoo, Makoto Osaki

**Affiliations:** 1 Department of Orthopedic Surgery, Nagasaki University Graduate School of Biomedical Sciences, 1-7-1 Sakamoto, Nagasaki, 852-8501 Japan; 2 Department of Health Sciences, Nagasaki University Graduate School of Biomedical Sciences, 1-7-1 Sakamoto, Nagasaki, 852-8520 Japan; 3 Department of Physical Therapy Science, Nagasaki University Graduate School of Biomedical Sciences, 1-7-1 Sakamoto, Nagasaki, 852-8520 Japan; 4 Department of Nursing, Fukuoka International University of Health and Welfare, 1-7-4 Momochihama, Sawara, Fukuoka 814-0001 Japan

**Keywords:** Aspiration pneumonia, Epidemiology, Emergency transportation record

## Abstract

**Background:**

Aspiration pneumoniae remains a major health concern, particularly in the older population and has poor prognosis; however, the concept itself remains vague worldwide. This study aimed to determine the actual situation and characteristics of aspiration pneumonia from 2005 to 2019 in Nagasaki Prefecture, Japan.

**Methods:**

Cases of aspiration pneumonia that occurred in the Nagasaki Prefecture between 2005 and 2019 were analyzed using emergency transportation records. The number of occurrences and incidence were analyzed according to age, sex, month, day of the week, and recognition time to clarify the actual situation of aspiration pneumonia.

**Results:**

The total number of new aspiration pneumonia cases was 8,321, and the mean age of the patients was 83.0 years. Annual incidence per 100,000 population increased from 12.4 in 2005 to 65.1 in 2019, with the most prominent increase in the ≥ 80-year-old stratum. Males (55.1%) were more commonly affected than females (44.9%), and 82.2% of the cases involved patients aged ≥ 70 years. No significant correlations were observed between the incidence of aspiration pneumonia and season, month, or day of the week. Aspiration pneumonia occurred frequently in houses (39.8%) and facilities for elderly individuals (40.8%). At 7 days after admission, 80.9% of patients were still hospitalized and 6.5% had died.

**Conclusions:**

The incidence of aspiration pneumonia with risks of severity and mortality is increasing among elderly individuals. Valid preventive measures are urgently needed based on the findings that the disease occurs in both household and elderly care facility settings, regardless of the season.

**Supplementary Information:**

The online version contains supplementary material available at 10.1186/s12890-024-03015-8.

## Key points

- The annual incidence of aspiration pneumonia per 100,000 population increased from 12.4 in 2005 to 65.1 cases in 2019 in the Nagasaki Prefecture.

- Approximately 82% of the patients were aged ≥ 70 years, and the incidence was frequent in houses (39.8%) and facilities for elderly individuals (40.8%).

- After one week, a majority of patients (80.9%) required inpatient treatment, and 6.5% of patients died.

**Supplementary Information:**

The online version contains supplementary material available at 10.1186/s12890-024-03015-8.

## Why does this matter?

The increasing number of cases of aspiration pneumonia among older adults is a cause for concern in Japan. Currently, an increasing number of clinical research is investigating its characteristics and patterns of occurrence. This study provides comprehensive insights into the increase in the number of cases over the years, settings in which the disease most often occurs, and the most affected age groups in the Nagasaki Prefecture in Japan. This study will help to better prepare against aspiration pneumonia.

## Background

The number of patients with pneumonia is increasing with an increase in the population of older adults, and aspiration pneumonia accounts for the majority of cases [1], especially in people aged ≥ 65 years [2–4]. Patients with dysphagia are more likely to develop pneumonia at a rate of 1.6–11.9 times greater than patients without dysphagia [1, 5–7]. Taylor et al. [5] revealed that the 30-day mortality from pneumonia in patients at risk of aspiration was 17.2% when compared with 7.7% in patients not at risk. Therefore, in the 2017 Ministry of Health, Labor, and Welfare report, “aspiration pneumonia” was added as a category of cause of death [8]. The number of deaths owing to aspiration pneumonia was 35,740 (mortality rate, 28.7%), with the disease ranking seventh among the causes of death [8]. A meta-analysis conducted in Japan in 2016 reported that patients with aspiration pneumonia had poorer short- and long-term prognoses than those with other types of pneumonia [9]. Even if the disease does not lead to death and is cured, a decline in physical function and an increase in the amount of assistance required in daily activities are inevitable [3, 5, 6, 9, 10]. Aspiration pneumonia constitutes one of the lung diseases associated with dysphagia [1–3, 5, 6]. The 2017 Japanese Respiratory Society’s guidelines for the management of pneumonia in adults define it as “pneumonia that occurs in cases in which dysphagia and aspiration have been proven (or are strongly suspected) [11].” However, the concept itself remains vague worldwide, and global awareness is lacking; thus, neither diagnostic nor treatment strategies have been clearly established [12, 13]. Therefore, understanding the pathology of aspiration pneumonia, early diagnoses, and establishing an appropriate treatment system are urgent clinical issues.

The Japanese Respiratory Society recommends pneumonia treatment by dividing it into community-acquired pneumonia (CAP), hospital-acquired pneumonia (HAP), and nursing and healthcare-associated pneumonia (NHCAP). Although guidelines have been published, several aspects of aspiration pneumonia overlap with those of HAP or NHCAP owing to patient characteristics, and the actual situation of aspiration pneumonia remains largely unknown. Teramoto et al. [2] conducted a multicenter prospective study at 22 Japanese centers from 2004–2005. Aspiration pneumonia was reported in 394 (66.8%) of the 589 total cases of pneumonia; however, most epidemiological findings in previous studies constituted aggregated data on a facility-by-facility basis [2, 3, 5, 9]. Therefore, the number of patients is small, and the accuracy of the incidence rate in a limited area and the various factors associated with the disease cannot be sufficiently investigated.

In Japan, all ambulance services are required by law to record prehospital transport data, such as age, sex, address, and recognition time (the time at which the ambulance was called) and hospital arrival. Nagasaki Prefecture launched the Nagasaki Practical Emergency Medical Liaison Committee in 1988 and introduced an original emergency transportation record system. The format was unified in 2004 and included additional information such as diagnostic codes, definitive diagnosis, and outcome at 1 week after ambulance transport. Overall, 13 main diagnostic codes are used by physicians. The average collection rate of the record has been reported to be 91.6%–93.1% [14, 15]. This high collection rate of emergency transportation records offers accurate and reliable objective data and enables high-quality analysis of severe disease and trauma in a specific region. The aim of the present study was to elucidate the current incidence and trends of aspiration pneumonia in the Nagasaki Prefecture from 2005 to 2019 and clarify the characteristics of aspiration pneumonia using emergency transportation records.

## Methods

### Study design and data collection

We applied for permission to use data from the Nagasaki Prefecture emergency transportation records to the Nagasaki Prefecture Medical Policy Division and the Nagasaki Prefecture Medical Control Council and received approval from the Review Committee (27 Medical Policy No. 876). The total number of emergency transports from 2005 to 2019 was 825,089, and the mean collection rate of emergency transportation records was 93.3% (*n* = 769,799). The inclusion criteria for this study were as follows: aspiration pneumonia as a definitive diagnosis. The definitive diagnosis was made by the attending physician at 1 week after ambulance transport. We excluded cases with no definitive diagnosis, no description of outcome, or unknown type of disease in emergency transportation records. Cases involving secondary transportation to another tertiary hospital and those with unknown transport type were also excluded.

The parameters to be analyzed included date and time of ambulance transport, sex (male or female), age (5-year strata), location of occurrence (indoors vs. outdoors), and outcomes at 1 week after ambulance transport, including status and transferred hospital. The total number of cases per year and incidence of cases in each study year were calculated according to the number of cases per 100,000 population based on the Nagasaki Prefecture census [16]. The initials and date of birth were omitted from the database information to ensure the protection of personal information. Therefore, duplication of cases was checked based on ambulance number, date, and time of ambulance transport. The location of occurrence was categorized as “house,” “hospital and clinic,” “nursing home (special care, group home, or others)” and “elderly care facilities.” In addition, we collected information on the day of the week, recognition time, and outcome 1 week later. Status at 1 week after ambulance transport was divided into five categories: “hospitalization,” “transfer to a different hospital,” “discharge from the hospital in an ambulatory state,” “mortality,” and “unknown.” The present study was conducted with the approval of the research ethics committee at Nagasaki University Graduate School of Biomedical Sciences (Approval number: 16031085).

### Statistical analysis

The categories/ratios of variables such as outcomes and treatment history at 1 week after ambulance transportation were compared using Pearson’s Chi-square test. Continuous variables such as age strata and monthly and daily variations were tested with one-way analysis of variance multiple comparison tests followed by post hoc Tukey–Kramer and Bonferroni/Dunn multiple comparison tests. All data were analyzed using SPSS version 22.0 (SPSS, Chicago, IL, USA). Values are expressed as mean ± standard deviation. Statistical significance was defined at *P* < 0.01.

## Results

### Number and incidence rate of cases

The total number of cases of new aspiration pneumonia, sex, and total population including the number of individuals aged ≥ 65 years in the Nagasaki Prefecture in each year are summarized in Table 1. The total number of cases of new aspiration pneumonia between 2005 and 2019 was 8,321, and it tended to increase gradually, whereas the population decreased yearly. Consequently, the incidence tended to increase gradually from 12.4 per 100,000 population per year in 2005 to 65.1 per 100,000 population per year in 2019.

**Table 1:** Population trends in the Nagasaki Prefecture and incidence of aspiration pneumonia

Year	Total population	Population of individuals aged ≥ 65 years	Aging population percentage(%)	Aspiration pneumonia	Incidence per 100,000	Male/Female ^a^
2005	1,478,632	348,820	23.6	183	12.4	106/72
2006	1,466,512	355,228	24.2	176	12.1	91/78
2007	1,453,740	361,143	24.8	206	14.2	107/95
2008	1,441,451	364,438	25.3	232	17.3	124/99
2009	1,432,236	369,387	25.8	416	29.0	227/186
2010	1,426,779	369,290	25.9	429	30.1	220/190
2011	1,417,282	368,942	26.0	474	33.5	252/210
2012	1,407,925	377,759	26.8	570	41.7	296/258
2013	1,396,481	386,813	27.7	675	49.2	362/302
2014	1,385,533	397,260	28.7	681	49.4	357/318
2015	1,377,187	404,686	29.4	755	56.0	425/325
2016	1,366,514	412,690	30.2	858	63.2	449/392
2017	1,353,550	419,253	31.0	875	65.2	484/376
2018	1,339,438	423,907	31.6	938	71.3	531/388
2019	1,325,205	427,988	32.3	853	65.1	456/375

^a^Excluding 167 unknown cases

### Age- and sex-specific patient totals

**Fig. 1:**
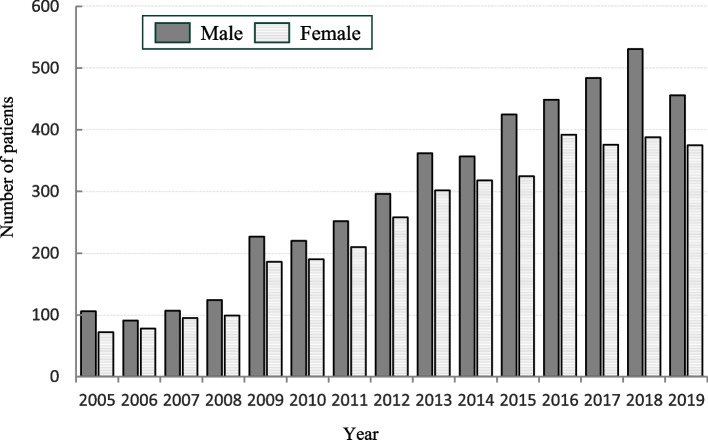
Annual change in the age- and sex-specific number of cases of aspiration pneumonia

**Fig. 2:**
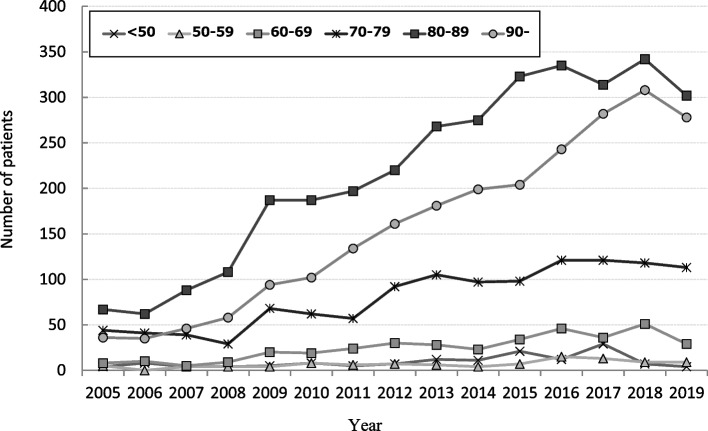
Annual trends of patients by age group

There were 4490 males, 3664 females, and 167 unknown cases, with a male-to-female ratio of 11:9. The mean age at onset was 83.0 ± 12.4 years, with that of male being 81.0 ± 12.1 years and that of female being 85.5 ± 12.1 years. The disease occurred more often in males in all years, and the incidence in both males and females showed an overall increasing trend. However, the number of females has plateaued since 2016 (Fig. 1). There were extremely few patients under the age of 50 years, and those above the age of 70 years accounted for 82.2% of the total. In addition, considering the trends over time by the age group, the increase rate was particularly remarkable among those aged ≥ 80 years (Fig. 2).

### Monthly and daily variations

**Fig. 3:**
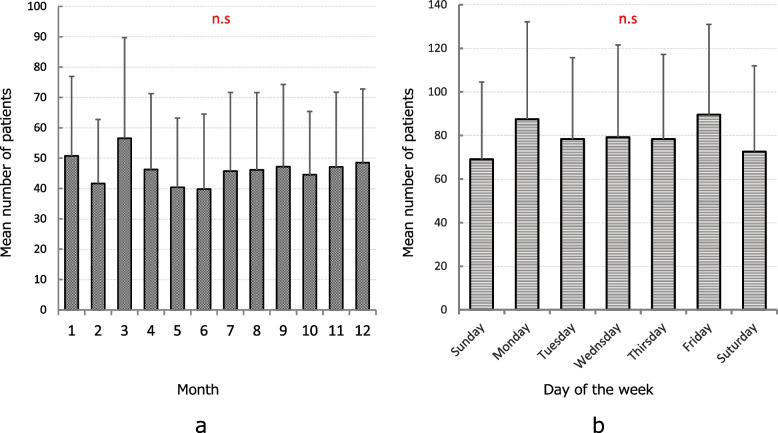
Average number of occurrences by month (**a**) and day of the week (**b**). Graphical representation: error bars are standard deviations. Analysis: One-way ANOVA, Bonferroni/Dunn multiple comparison test. n.s: Not significant

Figure 3 shows the average number of occurrences in each month and on each day of the week. There was no significant difference in the average number of cases for each month (*P* = 0.90), and cases occurred throughout the year regardless of the season (Fig. 3a). The average number of cases tended to be slightly higher on Mondays and Fridays, and relatively lower on the weekends (Saturdays and Sundays); however, no significant difference was observed in this regard (*P* = 0.77, Fig. 3b).

### Variations in the recognition time of day

Recognition time (the time at which the ambulance was called) was divided into 24 timeframes, and the mean number of patients per year was distributed (Additional File 1). The number of ambulance calls was relatively fewer from night to early morning, but relatively higher during daytime, especially from 9 to 11 am.

### Areas and places of occurrence

The number and percentage of sites of occurrence are summarized in Table 2. The highest number of cases of aspiration pneumonia occurred in houses (39.8%), followed by hospitals and clinics at 16.5%, nursing homes (special care) at 14.8%, and nursing homes (others) at 13.5%. However, the occurrence rate at facilities for elderly individuals as a whole was 40.8%, almost the same as that at houses. Most cases of aspiration pneumonia occurred indoors, with 91.5% occurring in living rooms, including hospital rooms and bedrooms, followed by entrances and corridors at 5.7%. Bathrooms, including dressing rooms, accounted for 1.6%, and stairs, toilets, and kitchens accounted for less than 1% each.

**Table 2:** Location by facility and place indoors

Area (*n* = 2,888)	n (%)	Place indoors (*n* = 1,152)	n (%)
House	1,150 (39.8)	Living room	1,054 (91.5)
Hospital and clinic	477 (16.5)	Entrance, Corridor	66 (5.7)
Nursing home (special care)	427 (14.8)	Bathroom, dressing room	19 (1.6)
Nursing home (others)	390 (13.5)	Kitchen	4 (0.4)
Elderly care facilities	221 (7.7)	Stairs	2 (0.2)
Group home	140 (4.8)	Toilet	3 (0.3)
Not indicated	83 (2.9)	Others	4 (0.4)

### Outcomes and treatments at 1 week after ambulance transport

At 1 week after ambulance transport, 80.9% of patients with aspiration pneumonia remained hospitalized for inpatient treatment and 9.6% were transferred to another hospital within 1 week after ambulance transport. Additionally, 2.9% of patients were discharged from the hospital in an ambulatory state, whereas mortality occurred in 6.5% of patients. In total, 1.4% of patients were surgically treated within 1 week after ambulance transport. The remaining 98.6% of patients received conservative treatment (Additional File 2).

Furthermore, 90.5% of patients were still hospitalized (80.9% of the patients were treated in the receiving hospital and 9.6% were transferred to another hospital) 1 week after ambulance transport.

## Discussion

Japan has the largest population of older adults worldwide, and its disease structure is changing. In 2017, the Japanese Respiratory Society published the “Japanese Respiratory Society Guidelines for the Management of Pneumonia in adults” [11] by integrating guidelines for CAP, HAP, and NHCAP. However, regarding aspiration pneumonia, only a list of risk factors is available, and almost no epidemiological evidence has been shown to elucidate the actual situation. This study provides valuable epidemiological knowledge by analyzing the incidence of aspiration pneumonia from emergency transportation record forms in the Nagasaki Prefecture.

Previous research examining the incidence of aspiration pneumonia in a specific limited area cannot be found. Our study revealed the actual number and incidence of aspiration pneumonia in the Nagasaki Prefecture from 2005 to 2019. A major strength of this study is that we analyzed large-scale data from original emergency transportation records, which have a high collection rate (93.1%) and include additional information at 1 week after ambulance transport. This methodology can supply reliable, objective data and enable high-quality analysis within a specific region. As a result, the annual number and incidence of aspiration pneumonia increased gradually since 2005 and reached 65.1 cases per 100,000 people in 2019. Nagasaki Prefecture, with an area of approximately 4,131 km^2^ and a population of approximately 1,325,205 in 2019, is located in the southwestern part of Japan. The population of residents aged > 65 years has been increasing and reached 427,988 in 2019, accounting for 32.3% of the total population. The aging population is considered as a major factor leading to an increase in the incidence of aspiration pneumonia, which often occurs in elderly individuals [3, 17–19], over time. Moreover, as the incidence in this study was calculated from the data of emergency transportation records, the potential incidence of aspiration pneumonia is estimated to be even higher. Thus, Nagasaki Prefecture can be regarded as a model of future population structures for developed countries, including Japan. Therefore, the medical significance of aspiration pneumonia, which has a high risk of leading to the requirement of nursing care or causing death, will increase in the near future.

Age- and sex-specific data indicated that aspiration pneumonia is approximately 1.2 times more common in males (55.1%) than in females (44.9%), and most of the cases occurred in patients aged ≥ 70 years (82.2%). The mean age at onset was 83.0 years (81.0 years for males and 85.6 years for females). Aspiration pneumonia is more common in people aged ≥ 75 years and in males, which is consistent with the results of several previous studies [2, 3, 19]. Teramoto et al. [2] reported that 80.1% of cases (306 of 382 cases) of pneumonia occurring in people aged ≥ 70 years were aspiration pneumonia. Furthermore, although there are more males aged < 90 years, the number of females with aspiration pneumonia tends to increase among people aged > 90 years, which is thought to reflect the male–to-female ratio in the population of older people aged > 90 years. Furthermore, the changes over time by age group indicate that the increase rate is particularly remarkable among those aged ≥ 80 years. The short- and long-term prognosis after aspiration pneumonia is poor [3, 5, 9, 10], with a 1-month mortality rate of 17.2% [5] and a 3-month mortality rate of 38.6% [3]. Therefore, in countries where the elderly population is expected to increase, it is important to enhance effective preventive measures in anticipation of the increase in patients with aspiration pneumonia.

To the best of our knowledge, no previous studies have demonstrated the number of cases of aspiration pneumonia by month or day of the week. The present study revealed that there were no significant differences in the number of cases of aspiration pneumonia by month or day of the week. Unlike epidemic pneumonia caused by bacterial or viral infections, aspiration pneumonia develops when secretions, such as food and saliva, are aspirated into the deep respiratory trachea owing to a decline in swallowing function. Thus, aspiration pneumonia is thought to occur at a constant rate, regardless of the season or day of the week. In contrast, the count of emergency calls was relatively low from night to early morning and became frequent during the daytime. Aspiration is classified into overt aspiration and silent aspiration depending on the presence or absence of coughing or choking. Aspiration pneumonia is typically caused by silent aspiration without these symptoms [20]. As silent aspiration includes microaspiration that occurs during sleep [21, 22], it is speculated that a considerable number of aspirations also occur at night. However, elderly people, who often develop aspiration pneumonia, do not show typical symptoms and are less aware of it. In particular, in patients with cerebrovascular or central nervous system disorders, there is a high possibility that surrounding caregivers may notice abnormalities and call for an ambulance. Consequently, the recognition of cases may have increased during the daytime, especially around 9 to 11 am.

No previous study has focused on the location of occurrence and outcome after 1 week in the area that was covered in this study. Regarding the location of occurrence, “house” (39.8%) was the most common, followed by “hospital and clinic” at 16.5%, “nursing homes (special care)” at 14.8%, and “nursing homes (others)” at 13.5%. In terms of indoor locations, 91.7% of the incidents occurred in living rooms, patient rooms, bedrooms, and other living rooms. Generally, it is considered that patients who develop aspiration pneumonia are elderly individuals, have many comorbidities, and repeatedly experience aspiration owing to a decline in swallowing function [23, 24]. In other words, aspiration pneumonia is more likely to occur in terminally ill or elderly people who are in nursing homes or who are almost bedridden in their own rooms. The present study found that almost 90.5% of patients were still hospitalized (80.9% of the patients were treated in the receiving hospital and 9.6% were transferred to another hospital) 1 week after ambulance transport. This observation indicates that most cases of aspiration pneumonia require hospitalization for 1 week or more. In the early stages of pneumonia owing to aspiration, there are only a few symptoms or only mild systemic symptoms such as a mild fever, fatigue, and loss of appetite. Nonetheless, in cases with immune dysfunction, infection could expand and lead to severe pneumonia [1, 3]. The Adult Pneumonia Treatment Guidelines also recommend that a treatment strategy should be formulated based on a comprehensive assessment of the risk of resistant bacteria, general condition (terminal stage/senility), and severity. In this study, more than 90% of patients were hospitalized 1 week after ambulance transport and 6.5% of patients had a fatal outcome. Therefore, it is necessary to formulate a treatment strategy carefully considering the background factors.

Relying on the data from emergency transportation records in this study, several potential limitations must be considered. First, patients from the same institution or those admitted to the hospital by other means could not be counted, raising the possibility of duplicates owing to the same patient being transported via different ambulances on separate occasions. Second, as a methodological limitation, we could not ascertain the percentage of patients with aspiration pneumonia transported by ambulance out of the total number of patients. Moreover, considering the evolving awareness and diagnostic criteria for aspiration pneumonia over time, it is plausible that improvements in recognition and clarity could impact the accuracy of diagnoses. These limitations may introduce bias, and consequently, the results of this study could not directly identify the exact risk factors of aspiration pneumonia. We analyzed the actual situation of aspiration pneumonia occurring within a specific region using a relatively large number of objective data obtained from emergency transportation records with a high response rate. To the best of our knowledge, this is the first study to focus on aspiration pneumonia from an epidemiological perspective. Therefore, the findings of the present study may compensate for such deficits, and it is important to utilize these findings for developing effective preventive measures against aspiration pneumonia.

## Conclusions

The present study investigated 8,321 cases of aspiration pneumonia from 2005 to 2019 in the Nagasaki Prefecture using emergency transportation records, and analyzed the current incidence, trends, and characteristics. The mean patient age at occurrence was 83.0 years, and the annual incidence per 100,000 population increased from 12.4 in 2005 to 65.1 in 2019. Males (55.1%) were more commonly affected than females (44.9%), and 82.2% of cases involved patients aged ≥ 70 years. Aspiration pneumonia occurred at a constant rate regardless of the season or day of the week, and frequently in houses (39.8%) and facilities for elderly individuals (40.8%). At 7 days after admission, 80.9% of patients remained hospitalized, and 6.5% had died. The findings of the present study should be utilized to valid preventive measures against aspiration pneumonia.

### Supplementary Information

**Supplementary Material 1.**

**Supplementary Material 2.**

## Data Availability

The data that support the findings of this study are available from the Nagasaki Medical Control Council, but restrictions apply to their availability since the data were used under license for the current study, and are therefore not publicly available. The data are, however, available from the authors upon reasonable request and previous permission from the Nagasaki Medical Control Council.

## Abbreviations

CAP: Community-acquired pneumonia
HAP: Hospital-acquired pneumonia
NHCAP: Nursing and healthcare-associated pneumonia
