# Supplementary material for: Incidence and characteristics of aspiration pneumonia in the Nagasaki Prefecture from 2005 to 2019
Source: BMC Pulm Med. 2024 Apr 20;24:191. doi: 10.1186/s12890-024-03015-8 (PMC11032591; doi:10.1186/s12890-024-03015-8)
Supplement: Supplementary file 1 — Supplementary Material 1. [file 12890_2024_3015_MOESM1_ESM.zip › Additional file 1.docx]

**Additional files**

**Additional File 1**

**Recognition time**

Graphical representation: error bars are standard deviations.

Analysis: One-way ANOVA, Bonferroni/Dunn multiple comparison test

Aspiration pneumonia tends to occur more frequently at 09:00–11:00 am than at other timeframes

*: *P* < 0.01 compared with other timeframes, except 10:00–11:00 am

^§^: *P* < 0.01 compared with other timeframes, except 09:00–10:00 am
