# Supplementary figures and images for: Incidence and characteristics of aspiration pneumonia in the Nagasaki Prefecture from 2005 to 2019
Source: BMC Pulm Med. 2024 Apr 20;24:191. doi: 10.1186/s12890-024-03015-8 (PMC11032591; doi:10.1186/s12890-024-03015-8)

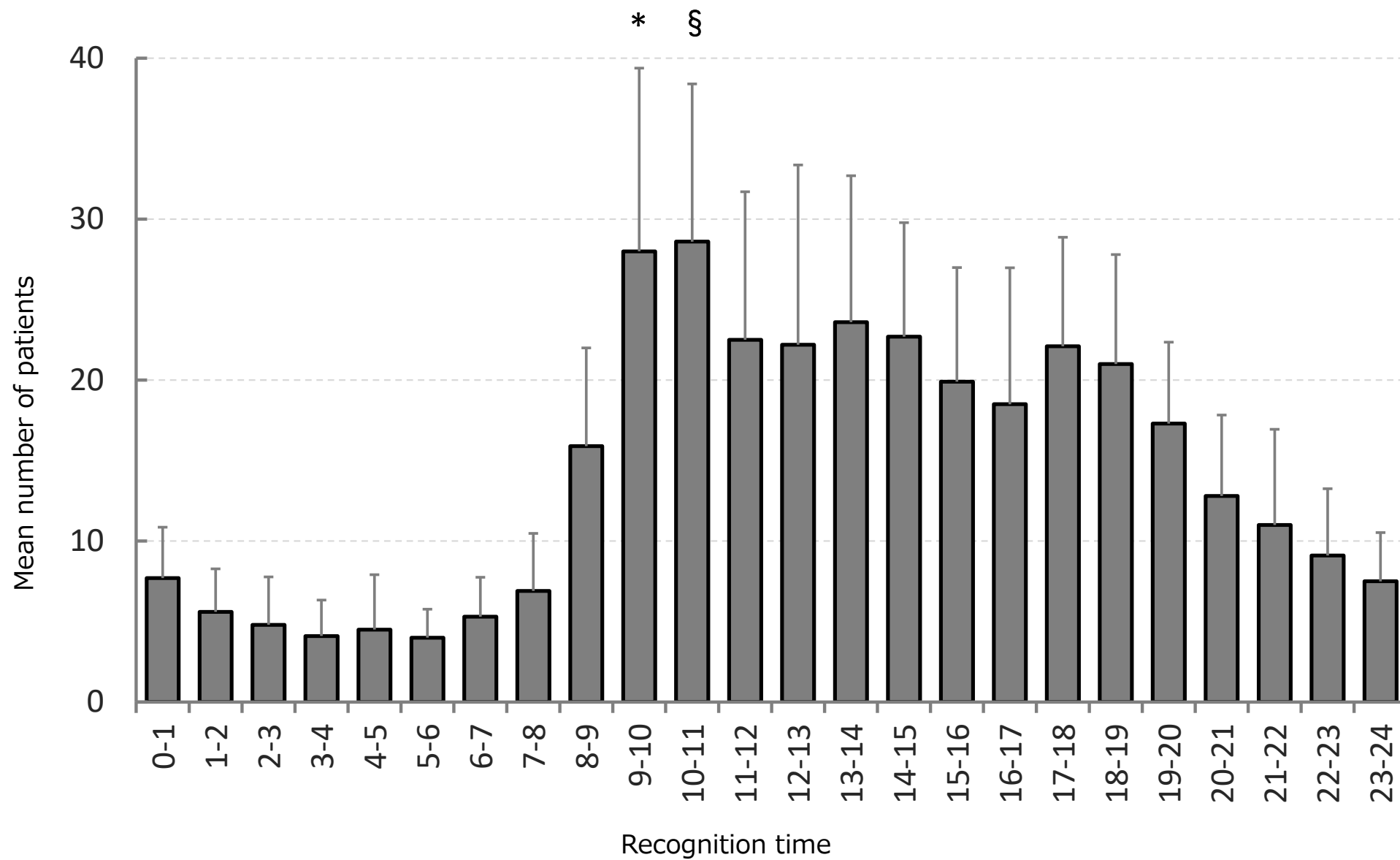

Additional File 1

Supplement: Supplementary file 1 — Supplementary Material 1. [file 12890_2024_3015_MOESM1_ESM.zip › Additional File 1.pdf]
