# Supplementary material for: Incidence and characteristics of aspiration pneumonia in the Nagasaki Prefecture from 2005 to 2019
Source: BMC Pulm Med. 2024 Apr 20;24:191. doi: 10.1186/s12890-024-03015-8 (PMC11032591; doi:10.1186/s12890-024-03015-8)
Supplement: Supplementary file 2 — Supplementary Material 2. [file 12890_2024_3015_MOESM2_ESM.docx]

**Additional File 2**

Outcome after 1 week

| Outcome 1 week after ambulance transport  (n=3,578) | n (%) | Treatment 1 week after ambulance transport (n=3,585) | n (%) |
| --- | --- | --- | --- |
| Hospitalization | 2,894 (80.9) | Surgery | 50 (1.4) |
| Transfer | 344 (9.6) | Conservative | 3,533 (98.6) |
| Mortality | 233 (6.5) |  |  |
| Ambulatory | 104 (2.9) |  |  |
| Unknown | 3 (0.1) |  |  |
